# Fukushima after the Great East Japan Earthquake: lessons for developing responsive and resilient health systems

**DOI:** 10.7189/jogh.07.010501

**Published:** 2017-06

**Authors:** Shingo Fukuma, Shahira Ahmed, Rei Goto, Thomas S Inui, Rifat Atun, Shunichi Fukuhara

**Affiliations:** 1 Department of Healthcare Epidemiology, Kyoto University, Yoshidakonoe, Sakyo, Kyoto 606–8501, Japan.; 2 Center for Innovative Research for Communities and Clinical Excellence (CIRC(2)LE), Fukushima Medical University, Fukushima, Japan; 3 Boston University School of Public Health, Department of Global Health, 801 Massachusetts Avenue, Boston, Massachusetts, USA; 4 Graduate School of Business Administration, Keio University, Yokohama, Japan; 5 Regenstrief Institute, Indiana University School of Medicine, Indianapolis, Indiana, USA; 6 Department of Global Health and Population, Harvard T.H. Chan School of Public Health, Harvard University, Boston, Massachusetts, USA; * Joint last authorship.

## Abstract

**Background:**

On 11 March 2011, the Great East Japan Earthquake, followed by a tsunami and nuclear–reactor meltdowns, produced one of the most severe disasters in the history of Japan. The adverse impact of this ‘triple disaster’ on the health of local populations and the health system was substantial. In this study we examine population–level health indicator changes that accompanied the disaster, and discuss options for re–designing Fukushima’s health system, and by extension that of Japan, to enhance its responsiveness and resilience to current and future shocks.

**Methods:**

We used country–level (Japan–average) or prefecture–level data (2005–2014) available from the portal site of Official Statistics of Japan for Fukushima, Miyagi, and Iwate, the prefectures that were most affected by the disaster, to compare trends before (2005–2010) and after (2011–2014) the ‘disaster’. We made time–trend line plots to describe changes over time in age–adjusted cause–specific mortality rates in each prefecture.

**Findings:**

All three prefectures, and in particular Fukushima, had lower socio–economic indicators, an older population, lower productivity and gross domestic product per capita, and less higher–level industry than the Japan average. All three prefectures were ‘medically underserved’, with fewer physicians, nurses, ambulance calls and clinics per 100 000 residents than the Japan average. Even before the disaster, age–adjusted all–cause mortality in Fukushima was in general higher than the national rates. After the triple disaster we found that the mortality rate due to myocardial infarction increased substantially in Fukushima while it decreased nationwide. Compared to Japan average, spikes in mortality due to lung disease (all three prefectures), stroke (Iwate and Miyagi), and all–cause mortality (Miyagi and Fukushima) were also observed post–disaster. The cause–specific mortality rate from cancer followed similar trends in all three prefectures to those in Japan as a whole. Although we found a sharp rise in ambulance calls in Iwate and Miyagi, we did not see such a rise in Fukushima: a finding which may indicate limited responsiveness to acute demand because of pre–existing restricted capacity in emergency ambulance services.

**Conclusions:**

We analyze changes in indicators of health and health systems infrastructure in Fukushima before and five years following the disaster, and explored health systems’ strengths and vulnerabilities. Spikes in mortality rates for selected non–infectious conditions common among older individuals were observed compared to the national trends. The results suggest that poorer reserves in the health care delivery system in Fukushima limited its capacity to effectively meet sudden unexpected increases in demand generated by the disaster.

On March 11th 2011, a massive earthquake, the Great East Japan Earthquake, followed by a tsunami, and tsunami damage–related nuclear–reactor meltdowns produced one of the most severe disasters in the history of Japan [1]. Among all of Japan’s prefectures, Fukushima was the most severely affected by this “triple disaster (earthquake, tsunami, and nuclear meltdown)” [2]. The adverse impact of the triple disaster on the health of local populations and on the health system was substantial, with destruction of infrastructure (including hospitals, clinics and emergency transportation), homes and lives.

In Fukushima, nearly 3770 people died in the “disaster”, and many of those deaths were caused by the tsunami [3]. More than 18 030 housing facilities were completely destroyed, and 75 159 were partially destroyed. The total cost of the damage to public facilities was estimated at 599.4 billion yen (equivalent to US$ 5.2 billion in 2017 exchange rates) [4]. At the time of the disaster, the Fukushima Daiichi nuclear power station was hit by a huge tsunami. The tsunami induced damage led to a series of events that triggered core meltdowns. Radioactive materials leaking from the plant forced people who had lived nearby to evacuate their homes. The Japanese government decided to restrict access to nearby areas and about 108 000 people were still considered to be displaced evacuees as of July 2015, including 63 000 inside Fukushima prefecture but not in their original homes. Faced with this surge of need and demand for health care, the capacity of health systems of Fukushima and the other most–affected nearby prefectures may have been exceeded. However, to date, no study has been undertaken to examine the effect of the triple disaster on the health system in Fukushima and the health system response.

This study examines changes in population–level health indicators before and after the triple disaster to ascertain the effect of the triple disaster on population health and the health system in Fukushima and surrounding prefectures, and discusses options for re–designing the health system to enhance its responsiveness and resilience to current and future shocks.

## METHODS

### Setting and data sources

We used publicly available data, data from government sources, and published literature for Japan overall and for Fukushima, Miyagi and Iwate – the prefectures that were most affected by the Great East Japan Earthquake. All three prefectures of Fukushima, Miyagi and Iwate are in the Tohoku region, which is known for being socio–economically less well developed compared to other regions of Japan.

Data for a predetermined list of population, health systems, and outcome indicators were collected for Japan overall (average) and prefectural–level aggregates, further delineated below. Our main source of data was the website of Official Statistics of Japan, managed by the National Statistics Center [4]. These data are officially compiled and aggregated from national surveys and administrative registers by the Japanese government and made available at the website on a quarterly or annual basis. We confined our study to time period 2005–2014 for which data were available for most indicators and to span the time before (2005–2010) and after (2011–2014) the disaster.

### Population indicators

To analyze contextual characteristics in Fukushima and other prefectures, we used demographic and socio–economic indicators, including: population size, population density, percentage of people over 65 years, percentage of productive population, fertility rate, real gross domestic product (GDP), unemployment proportion, job category, crime rate and number of evacuees due to the disaster. Those indicators were measured by surveys of the Japanese Ministry of Internal Affairs and Communications [5].

### Health system indicators

To assess health system factors that might affect mortality rates, we used the following supply–side indicators: number of hospitals, number of clinics, number of physicians, number of nurses, number of outpatient visits number of hospitalizations, number of ambulance calls, and health expenditure per capita. Those indicators were measured by Japanese Ministry of Health, Labour and Welfare surveys [6].

### Health outcome indicators

Our main health outcome indicators were cause–specific mortality rates, which are measured regularly through the survey of vital statistics in Japan [7]. The causes of death of greatest interest were: all–cause mortality, and that from myocardial infarction, cerebrovascular disease, cancer, lung disease, and suicide.

We reasoned a priori that cardiovascular mortality and stroke might be acutely reactive to the stress of the disaster, social and physical dislocations, as might suicide in the face of great personal and physical losses suffered. Similarly, marginally compensated chronic pulmonary disease and/or reactive airway disease might also respond to the altered circumstances imposed on the disease. Cancer mortality, however, might not show an acute change, since cancers might have a long premorbid phase. We used age–adjusted cause–specific mortality rates based on the model population of Japan in 1985 [8].

### Analyses

All quantitative data were analyzed using Stata v.13 (StataCorp. 2013. Stata Statistical Software: Release 13. College Station, TX: StataCorp LP, USA). We analyzed all indicators before and after the disaster to produce descriptive statistics and to establish a time–trend line plot to examine changes over time (2005–2014) and to compare the trends before (2005–2010) and after (2011–2014) the disaster in Japan (using Japan)–average, Fukushima, Miyagi and Iwate.

## RESULTS

### Changes in population indicators

**Table 1** displays demographic and socio–economic indicators before and after the triple disaster. Real GDP per capita in 2010, before the disaster, was 3.8, 3.5, 3.3 million yen in Fukushima, Miyagi and Iwate, respectively, compared to 4.0 million yen for Japan overall. Elderly over 65 years of age represented 25.0%, 22.3%, and 27.2% of the population respectively in Fukushima, Miyagi and Iwate compared to 23.0% in Japan overall. A smaller proportion of the population in each of the three prefectures participated in jobs in “high–level industry” (**Table 1**).

**Table1:** Population and health system indicators of Fukushima, Iwate, Miyagi and Japan before and after the earthquake*

Indicators	Fukushima		Miyagi		Iwate		Japan	
Before	After	Before	After	Before	After	Before	After
Population (100 000 people)	20.5	19.7	23.3	23.0	13.5	13.1	1270.6	1263.9
Population density (/km^2^)	147.2	141.2	322.3	319.5	87.1	84.8	343.4	341.3
Percentage of elderly over 65 (%)	25.0	26.9	22.3	23.8	27.2	28.7	23.0	25.1
Percentage of productive population aged 15–64 (%)	62.5	60.4	66.0	63.4	61.4	59.0	65.8	62.1
Fertility rate (per 1000 people)	8.0	7.5	8.2	8.2	7.4	7.2	8.5	8.2
Total fertility rate	1.49	1.53	1.25	1.34	1.37	1.46	1.37	1.43
Real GDP (trillion yen)	7.6	7.6	8.2	9.1	4.4	4.7	512.5	517.5
Real GDP per capita (million yen)	3.8	3.9	3.5	3.8	3.3	3.6	4.0	4.1
Unemployment proportion (%)	5.1	3.6	5.7	4.1	5.1	3.3	5.1	4.0
Percentage of job category:								
Primary industry (%)	7.6	–	5.0	–	12.0	–	4.0	–
Secondary industry (%)	29.2	–	22.1	–	24.3	–	23.7	–
Tertiary industry (%)	60.0	–	70.5	–	62.3	–	66.5	–
Crime rate (per 100 000 people)	6.7	5.3	10.0	10.1	5.9	6.0	11.0	11.1
Number of evacuees to the other areas in the same prefecture (per 1000 people)	–	60.6	–	53.9	–	24.7	–	190.5
Number of evacuees to the other prefectures (per 1000 people)		44.1		6.7		1.5		–

*Data before the disaster were measured in 2010. Data after the disaster were measured in 2012 (real GDP), 2015 (number of evacuees) or 2013 (other indicators). We extracted data from the portal site of Official Statistics of Japan [8].

In Fukushima, the relative decline in the population level after the disaster was greater than that observed in other prefectures: 3.9% between 2015 and 2010 compared to 1.29% in Miyagi, 2.9% in Iwate, and 0.53% in Japan overall. Compared to the trends observed in the rest of the country, the age structure in Fukushima is changing more rapidly, with an increasing proportion of people over the age of 65 years after the disaster reaching 26.9% of the total population in the prefecture in 2013, compared with 25.0% in 2010.

Fukushima’s economic indicators as measured by average real GDP and income per person remained flat after the triple disaster, but the industrial production index, which is used to track the production of manufacturing industries, declined, and by 2014 had not recovered to the pre–disaster levels achieved [9]. By contrast, in Miyagi, Iwate and Japan overall, average real GDP and income per person rose, while unemployment rates declined, between 2010 and 2012. Fukushima crime rates, which were already low compared to Japan as a whole, remained low and actually improved after the disaster.

By 2015, four years after the disaster, 190 000 people had remained as evacuees (located to prefectures all over Japan), and were unable to return to the coastal areas most affected by the disaster. This situation was worse for those from Fukushima, due to the nuclear power plant accident. Compared to Miyagi and Iwate, Fukushima had the highest number of evacuees residing in the same prefecture (60 600), and the highest number of evacuees located to other prefectures of Japan (44 100).

### Changes in health system indicators

All three prefectures studied were “medically underserved” before the disaster, with fewer physicians, nurses, ambulance calls and clinics per 100 000 residents compared with Japan averages.

**Figure 1:**
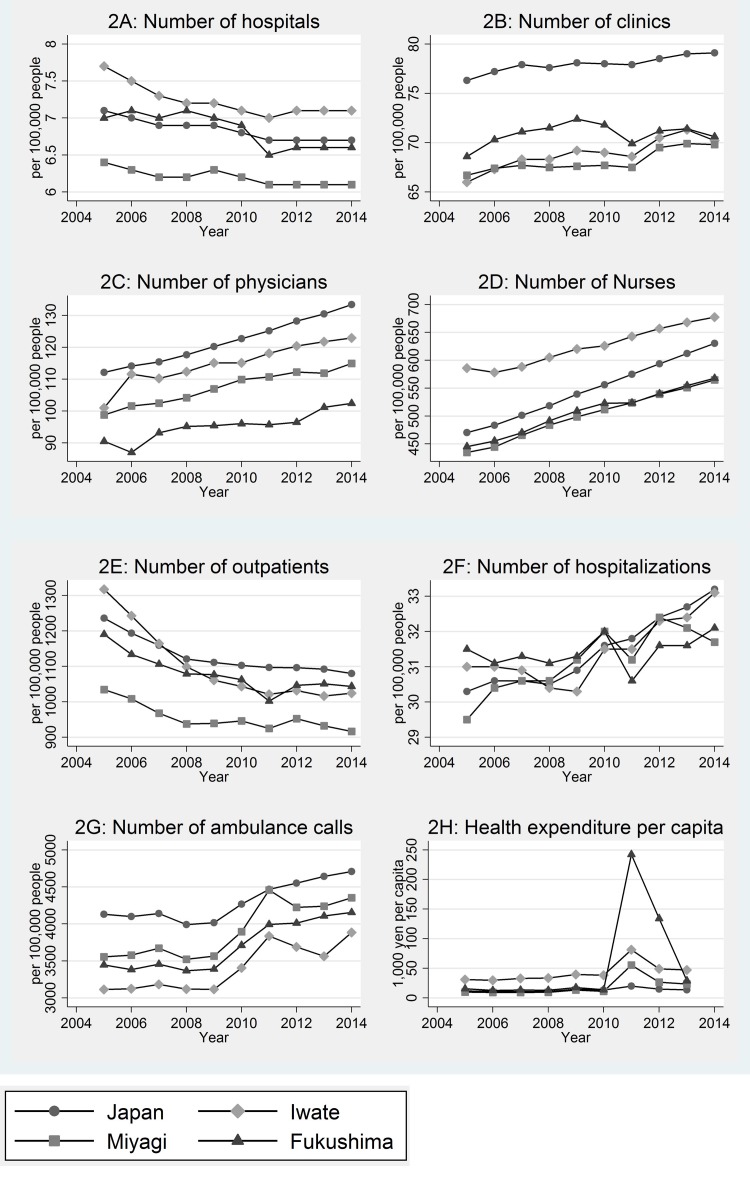
Time–trend in health system indicators 5 years after the disaster. 2A: Number of hospitals. 2B: Number of clinics. 2C: Number of physicians. 2D: Number of registered nurses. 2E: Number of outpatients. 2F: Number of hospitalizations. 2G: Number of ambulance call. 2H: Health expenditure per capita.

**Figure 1**shows time–trends for indicators related to health system capacity. The number of hospitals and clinics declined in Fukushima immediately after the disaster – a reflection of the physical destruction of facilities. The number of physicians and nurses in hospitals pre–disaster already was lower in Fukushima compared with the Japan–average (a deficit of 30 physicians/100 000 people and 120 nurses/100 000 people). Soon after the disaster, teams of health professionals from other prefectures were dispatched to the afflicted areas. Long–term efforts, however, are still needed to address the structural shortage of health care workers in Fukushima at present (see **Figure 1**, panels 2C and 2D).

In terms of service utilization in the health system, the number of outpatient visits and hospitalizations declined immediately after the disaster, especially in Fukushima and Miyagi. This decline may reflect the loss of medical facilities (hospitals and clinics). Health expenditures, however, rose dramatically in Fukushima just after the disaster. A breakdown of the rising health expenditures reveals that major capital investments related to reconstruction projects for environmental health (the construction of decontamination facilities needed to deal with radiation exposure) and expenditures for provision of medical care (repair and reconstruction of hospitals and clinics in coastal areas, as well as for recruitment of health care workers) constituted the main elements of the rise expenditures [9]. The number of ambulance calls increased gradually in Japan as a whole, a trend which may reflect the rising demand from increasing numbers of elderly patients in Japan’s aging society [10]. Although we found a sharp rise in ambulance calls in Iwate and Miyagi, we did not see such a rise in Fukushima, in spite of it having a higher proportion of older persons in the prefecture’s population compared to Iwate, Miyage and the rest of Japan. This ‘flattening’ in ambulance call rates in spite of an older population base might reflect the inability of the damaged emergency transportation system to respond to need.

### Changes in health outcome indicators

**Figure 2:**
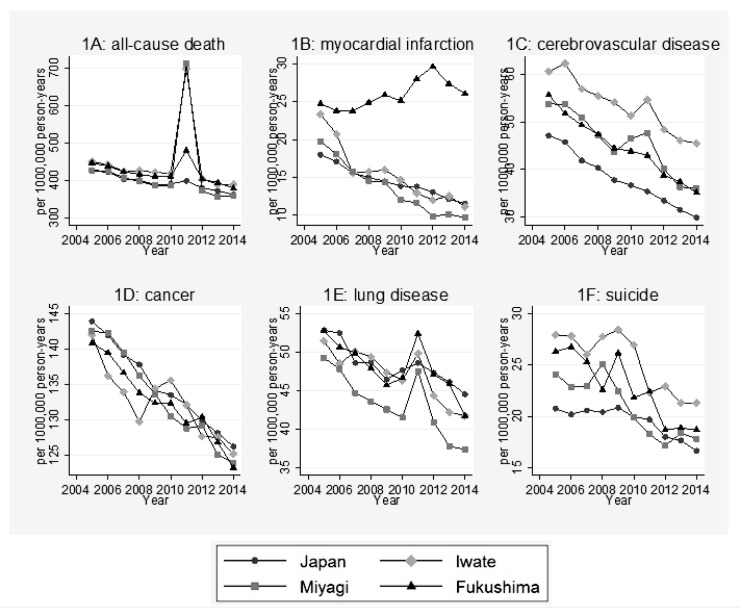
Time–trend in health outcome indicators 5 years after the disaster. 1A: Age–adjusted all–cause death rate. 1B: Age–adjusted death rate due to cardiovascular disease. 1C: Age–adjusted death rate due to cerebrovascular disease. 1D: Age–adjusted death rate due to cancer. 1E: Age–adjusted death rate due to lung disease. 1F: Age–adjusted death rate due to suicide.

**Figure 2** displays time–trends in health indicators before (2005–2010) and after (2011–2015) the disaster in Fukushima, Miyagi, Iwate and the average indicators for Japan as a whole.

Even before the disaster, age–adjusted all–cause mortality in Fukushima was in general higher than the national average for Japan. The mortality in Japan in 2010 was 390 deaths per 100 000 population while in Fukushima that rate was 415, rising to 480 in 2011, but decreasing to 403 in 2012. We found a higher rise in mortality in Iwate from 418 deaths per 100 000 population in 2010 to 699 in 2011and in Miyagi from 386 deaths per 100 000 population to 713 in 2011 after the disaster.

While other cause–specific mortality rates, such as deaths due to cancer, show similar trends in Fukushima to those in Japan as a whole, after the disaster the mortality rate due to myocardial infarction (MI) increased substantially in Fukushima, while this rate decreased nationwide. Mortality rate due to MI in Fukushima also differed from the rates and trends in Iwate and Miyagi where reductions in mortality rates from MI were evident (see **Figure 2**, panel 1B).

In Fukushima, Miyagi and Iwate there were also sharp increase in the rates of mortality from lung diseases in 2011 (**Figure 2**, panel 1E). While, nationally and in Fukushima there were steep declines in the mortality rates from lung diseases in the period 2005 to 2010, in Fukushima there was a sharp rise observed from 47 deaths per 100 000 in 2010 to 54 deaths per 100 000 in 2011. From 2012, in the three study prefectures the mortality rates form lung disease re–established their downward trend.

The suicide rates (**Figure 2**, panel 1F) in the three prefectures appeared to be declining before the disaster as well as in Japan as a whole. There appears to have been no ‘epidemic’ of suicides temporally related to the disaster in the three prefectures. By simple inspection, there may have been an excess of suicides in 2009, but not in 2011, when there may have been a sharper rate of decline in the three prefectures.

### Discussion

In the three affected prefectures of Fukushima, Miyake and Iwate in Japan, the Great East Japan Earthquake and its sequelae of a tsunami and nuclear reactor meltdown were responsible for major damage to persons and property. To our knowledge, this is the first paper to describe changes over time in multidimensional health and health system indicators for Fukushima and other affected prefectures in the period before (2005–2010) and five years after (2011–2014) the disaster.

By our observations, Fukushima, its residential populations, and those of its neighboring prefectures were already socio–economically and demographically vulnerable in 2011 to the destruction brought by the triple disaster. The health outcomes examined appear to show manifestations of the high burden of chronic conditions common in aging populations.

We found that mortality rate due to MI increased substantially in Fukushima, for example, while this rate decreased nationwide, but other cause–specific mortality rates such as deaths due to cancer show similar trends in Fukushima to those in Japan as a whole. Although we found a sharp rise in ambulance calls in Iwate and Miyagi, we did not see such a rise in Fukushima, which may indicate poor responsiveness of the health system in Fukushima, due to a limited capacity to respond to need/demand with emergency ambulance services. These results suggest that there were poorer reserves, and weaker emergency responsiveness and resilience of the health system in Fukushima than Iwate and Miyage. Hence, it was unable to meet the sudden and unexpected rise in demand for health services generated by the disaster.

Following the disaster, age–adjusted all–cause mortality in Fukushima and the two other prefectures affected by the disaster was higher than the average national rates. This difference could be attributed to health systems factors, such as poor quality of care and inadequate supply of resources in these prefectures, as well as the unique contextual factors in them (such as the socio–economic milieu), which might have magnified the adverse effects of the disaster, with the tsunami leading to widespread destruction of homes. Many people in the afflicted areas struggled with access to medications and treatments to effectively manage their chronic conditions, an adversity which could have resulted in excess premature deaths [11].

The observed high mortality rate due to MI in Fukushima may present a unique set of challenges for the health system in Fukushima. First, the shortage of physicians was more severe in Fukushima than in its neighboring prefectures. Second, and related to the first explanation, there were poorer reserves in health care delivery system as a whole in Fukushima, hindering an effective response to meet unexpected and sudden rise in demand generated by the disaster. Third, Fukushima has the third largest land–area among all prefectures in Japan, so it is likely that the time lapse for an effective response (for example as measured by ‘pain onset–to–balloon time’) for MIs was more likely to be longer than other prefectures. Fourth, the high rates of MI could reflect the changing demographic profiles in Fukushima, leading a relatively higher proportion of elderly residents as a result of younger and healthier people migrating out of the prefecture.

Mental health problems typically emerge after major disasters, but in the three prefectures affected by the disaster the suicide rates did not spike. It was reported that within days of the disaster there was a recognition by the government and local authorities of the psychological consequences of the events and ‘mental health care response teams’ were dispatched by the Ministry of Health, Labor and Welfare to the affected areas [12,13]. Risk factors for mental illness need continued attention, however [14]. A survey of self–reported mental health found that respondents felt a sense of “isolation” for at least 18 months after the disaster despite their participation in community–based programs [15].

There were also some striking and encouraging responses to the disaster, some from the health system, as it mobilized its remaining resources in unusual ways, and others from Japan’s civil society and cultural practices generally. The undersupply of health care workers, largely as a result of geographic maldistribution, is an important issue affecting Japan’s health system not only in Fukushima, but also elsewhere in rural Japan.

The aftermath of the triple disaster revealed social cohesion, as well as the strengths and deficiencies in the responsiveness and resilience of Japan’s health system. The societal response and resilience to the disaster was exemplary: not only did the social fabric not ‘tear,’ the society appears to have been able to weave a stronger fabric to protect its members, especially the elderly and vulnerable [16]. Community–level social cohesion before the disaster was shown to be associated with lower risk of post–traumatic stress disorder, and after the disaster social cohesion was maintained and strengthened to increase community resilience after the disaster [17]. Social violence, witnessed in other countries in the aftermath of natural disasters, did not emerge in the affected areas of Japan. Crimes did not increase, Conversely, we report that crime rates in Fukushima declined from 6.7 per 100 000 people in 2010 to 5.3 per 100 000 in 2012.

Advances in information communications technology (ICT) played an important role in local community as alternative information source and communication platform. Voluntarism was evident – providing much needed additional human resources. Yet, the responsiveness of the health system was challenged, and its resilience came under pressure, as the health system tried to meet the ongoing needs of vulnerable populations, in particular the elderly. Community resilience, which depends on local context and multilayered process [18], was evident in Fukushima.

Responsiveness challenges in the disaster were related to effective and timely integration of community and hospital responses, speed of communication, managing varied messages emerging from official sources and the media, transport – with consequent adverse effect on supply chain management for critical supplies – and the shortage of health human resources. There was strong public demand for high levels of transparency in relation to the course of events, timely communication and effective information dissemination.

Japan is rapidly aging and in terms of average life expectancy is ahead of other countries. Demographic shifts in the disaster–affected prefectures of Fukushima, Miyagi and Iwate, are particularly apparent, requiring an appropriate health system response to this unfavorable shift. Challenges to the health system brought by an aging of the population – such as disability and multimorbidity [19] – should be given priority in the future. In fact, population aging is, in itself, an internal shock to health system. Increased need for medical care and long–term care resources for the elderly population will be a major challenge for the re–design of sustainable health systems [20].

There are two significant limitations of this study. First, this is an ecological study using prefecture–level aggregated data. Socio–economic and demographics changed in the three study prefectures over time and those changes would affect both numerators and denominators in our analysis. For example, Fukushima is aging more rapidly with an increasing proportion of elderly people. This may be the result of many young families leaving Fukushima; for example, families who might be concerned about the long term effects of radiation exposure for their children. Second, we only used available data from the portal site of Official Statistics of Japan and were not able to secure individual level data on the affected and control populations. Third, the effect of the triple disaster on the health system of the affected prefectures may not be generalizable to other disasters. However, notwithstanding contextual differences, this unexpected major natural disaster revealed common problems for health systems that may be applicable to other prefectures of Japan. Even allowing for the methodological challenges faced by the study, reflecting on the lessons learned from Fukushima should be important when discussing options for re–designing health systems to enhance their responsiveness and resilience to major internal and external shocks.

In retrospect, several lessons emerge from the response of Fukushima to the triple disaster, ones that may inform health system transformations elsewhere to enhance responsiveness and resilience to shocks, but also in relation to managing wider social determinants and community aspects of disaster resilience. An expert group meeting in Fukushima under the auspices of the World Health Summit Regional Meeting in Japan in 2015 elaborated these lessons as follows [21]: Responsiveness can be enhanced by (i) establishing a local, regional and national framework for rapid information–sharing, decision–making and action; (ii) gathering timely information across sectors of government and industry for targeted action and dissemination to the public; (iii) creating sufficient reserves to rapidly mobilize and fill health system ‘gaps’ that emerge due to limited supply of critical resources and increased demand for resources immediately after a disaster; (iv) providing immediate access to transportation, communication, temporary shelter, clothing, and food to assure individual and population health security needs; (v) creating just–in–time management systems to deploy mobile heath teams and health workers in health systems; and (vi) integrating health system and social actions for a more comprehensive response. Resilience, on the other hand, the expert group concluded, can be developed and enhanced by (i) better monitoring the long–term effects of disasters, including mortality, disability, destitution, and social welfare in different population groups, especially the vulnerable, to inform current and future policies; (ii) establishing multi–sector action plans involving public agencies and the private sector; (iii) enabling community mobilization through social networks and building social capital; and (iv) developing and strengthening leadership at all levels of the health system to improve communication and inclusive decision making.

Fukushima illustrates the challenges faced by health systems in Japan and other countries globally, which are subject to rapidly changing contexts – as a result of swift demographic and epidemiological transitions (leading to population aging and a rapid rise of in the burden of chronic illness and disability), economic crises, ecological shocks from natural disasters and changing socio–cultural milieu – and have to respond and be resilient to the emerging challenges and shocks, while continuing to provide effective universal health coverage [22,23].

Contextual shocks and major disasters could happen anytime and anywhere worldwide, and their impact on health systems and health are globally relevant. Given the uncertainties, nothing less than transformative change is needed to create health systems in Japan and globally that are responsive and resilient to future shocks and emerging contextual challenges, including the rapid aging of our societies and the multimorbidity and disability this transition brings [18]. The Fukushima triple disaster is not the first, and will not be the last such challenge we face globally. Learning from our experience must be the order of the day.
